# The Use of Closed Incision Negative Pressure Therapy on the Medial Thigh Donor Site in Transverse Musculocutaneous Gracilis Flap Breast Reconstruction

**DOI:** 10.3390/jcm11102887

**Published:** 2022-05-20

**Authors:** Laura Cosima Siegwart, Christian Tapking, Yannick Fabian Diehm, Valentin Felix Haug, Amir Khosrow Bigdeli, Ulrich Kneser, Dimitra Kotsougiani-Fischer

**Affiliations:** Department of Hand, Plastic and Reconstructive Surgery, Microsurgery, Burn Center, BG Trauma Center Ludwigshafen, Hand and Plastic Surgery, University of Heidelberg, 67071 Ludwigshafen, Germany; lauracosima.siegwart@bgu-ludwigshafen.de (L.C.S.); christian.tapking@bgu-ludwigshafen.de (C.T.); yannick.diehm@bgu-ludwigshafen.de (Y.F.D.); valentin.haug@bgu-ludwigshafen.de (V.F.H.); amir.bigdeli@bgu-ludwigshafen.de (A.K.B.); ulrich.kneser@bgu-ludwigshafen.de (U.K.)

**Keywords:** closed incision negative pressure therapy, CINPT, transverse musculocutaneous gracilis flap, TMG flap, donor site morbidity, surgical site complications, breast reconstruction

## Abstract

The objective of this study was to examine the impact of closed incision negative pressure therapy (CINPT) on donor site complications and patient perceptions in transverse musculocutaneous gracilis (TMG) flap breast reconstruction. Our institution conducted a retrospective cohort study, including all patients with TMG flap breast reconstruction from 1 January 2010 to 31 December 2021. Patients were grouped according to conventional wound management or CINPT. Outcomes were surgical site complications, fluid drainage, time to drain removal, and in-hospital stay length. A patient survey was created. A total of 56 patients with 83 TMG flaps were included (control group: 35 patients with 53 TMG flaps; CINPT group: 21 patients with 30 TMG flaps). Patient characteristics were similar in both groups. The flap width was significantly larger in the CINPT group (8.0 cm vs. 7.0 cm, *p* = 0.013). Surgical site complications were reduced in the CINPT group without statistical difference (30.0% vs. 50.9%, *p* = 0.064). Fluid drainage and time to drain removal were similar in both groups. The average in-hospital stay was significantly shortened in the CINPT group (10.0 days vs. 13.0 days, *p* = 0.030). The survey excluded pain, skin irritations, and discomfort during sleep and movement in the CINPT group and showed that the patients felt well protected. This study fails to provide compelling evidence for CINPT to enhance incision healing on the donor site in TMG flap breast reconstruction. There was a trend toward reduced surgical site complications on the donor thigh and the in-hospital stay was shortened. Prophylactic CINPT increases patient comfort and provides a feeling of additional wound protection.

## 1. Introduction

Free flap breast reconstruction is considered the gold standard for post-mastectomy breast reconstruction, with success rates ofabove 98% [1,2]. Various free flaps enable plastic surgeons to individualize microsurgical breast reconstruction to patients’ preconditions and demands [3].

First introduced in 1992 by Yousif et al. and popularized by Schoeller and Wechselberger et al. for breast reconstruction in a series of publications since 2001 the transverse musculocutaneous gracilis (TMG) flap from the medial thigh has become a valuable choice for uni- and bilateral breast reconstruction for women with a low or normal body mass index (BMI), or those with limited soft tissue excess on the lower abdomen donor site [4,5,6,7].

The TMG flap is appreciated for its reliable anatomy, convenient surgical procedure, low donor site morbidity, and the appealing look of the reconstructed breast [8,9]. However, harvesting the TMG flap, including a horizontal soft tissue island and part of the gracilis muscle, creates an extensive wound on the medial thigh. The donor site is closed in primary intention similar to a cosmetic thigh lift. However, patient-related and surgery-related risk factors make the closed incision line prone to serious surgical site complications. Risk factors include previous breast cancer therapy, extensive tissue mobilization, distracting forces that pull apart from the suture line and bacterial contamination due to its close location to the intimate area. Thus, it is not surprising that wound breakdown, seroma, hematoma, and infection evolve in 0% to 26%, 0% to 15.5%, 0% to 12.5%, and 0% to 5% on the medial thigh donor sites on average [2]. Closed incision negative pressure therapy (CINPT) was specifically designed to manage closed incision lines with reduced healing capacities and could be advantageous to enhance incision healing on the donor site in TMG flap breast reconstruction. The concept is based on the protection of the clean wound environment, thediminishment of tension forces, the enhancement of tissue perfusion and the elimination of wound fluid [10,11,12]. The advantages of CINPT have been outlined in multiple studies in plastic and reconstructive surgery as well as in oncological breast surgery [13,14,15,16]. In particular, evidence exists for the superiority of CINPT compared to conventional wound management at the donor site of patients undergoing free flap breast reconstruction from the lower abdomen [17,18,19,20]. This is the first study to evaluate the use of CINPT on donor site healing and patient perception in TMG flap breast reconstruction.

## 2. Materials and Methods

Following ethical committee approval (2021-15766), a retrospective cohort study was conducted, including all patients who received TMG flap breast reconstruction in our institution (1 January 2010 to 31 December 2021) following successful breast cancer therapy or prophylactic mastectomy. Patients were grouped according to the incision management using conventional wound dressings (control group) or CINPT (CINPT group) on the medial thigh donor site. The electronic inpatient hospital information system was used for data acquisition. Patients’ characteristics [gender, age, BMI, smoking status, medical preconditions and previous breast cancer therapy], intraoperative data [microsurgical procedure, operation time, wound management], and outcomes were abstracted. Primary outcomes included non-operative and operative surgical site complications, daily and total fluid rate, time to drain removal, and in-hospital stay. In addition, patient-reported outcomes were surveyed in the CINPT group before discharge. Non-validated 5-point Likert scale questions were designed to survey skin irritations, pain, patient comfort during night sleep, mobility, and patient-perceived protection at the donor site. In addition, patients were asked whether they would choose CINPT in future again (yes/no). The postoperative follow-up was at least two months.

### 2.1. Closed Incision Management

**Figure 1:**
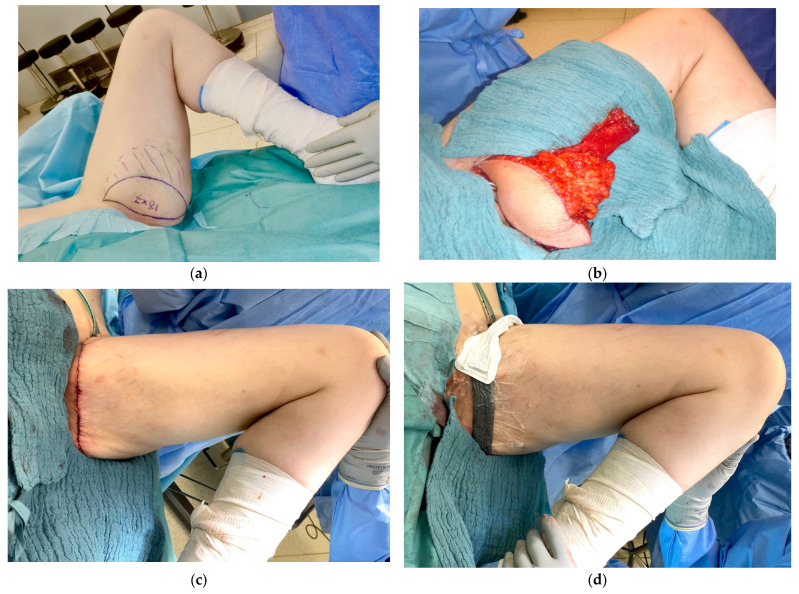
Intraoperative view of a normal-weight female patient (42 years, BMI 23.8 kg/m^2^) undergoing TMG flap harvest from the left thigh for free flap reconstruction of the right breast following successful breast cancer therapy. The patient suffered from invasive ductal carcinoma. In a previous surgery, nipple-sparing mastectomy was performed and an expander was placed in the right breast. (**a**) Patient in the supine position. Left leg in frog position with preoperative markings of the TMG flap outlining the skin island in average dimension (7 × 18 cm) and the subcutaneous fat extension to boost up flap volume. The skin island is limited to the medial aspect by the femoral neurovascular bundle and to the inferior part by a pinch grip. (**b**) TMG flap including the proximal skin island, the beveled fat extension, and part of the gracilis muscle. (**c**) Multi-layer closure of the donor site wound on the medial thighsimilar to a horizontal thigh lift. (**d**) CINPT on the closed suture line on the medial thigh donor site. The closed incision was covered with fatty gauze and customized black polyurethane foam. The transparent adhesive film was placed on the foam and small part of the surrounding skin. The soft port was placed and continuous negative pressure was applied.

TMG flap breast reconstruction was performed according to an in-hospital standardized protocol for both groups by senior board-certified surgeons in our institution. The surgical procedure was published in detail [8]. Donor site closure was performed in three layers with absorbable suture material. Firstly, a few inverted interrupted sutures were performed for the reduction in the dead space of the deep soft tissue (Vicryl 0, Ethicon, Norderstedt, Germany). Secondly, inverted interrupted sutures were used for the closure of the subcutaneous tissue (Monocryl 2.0, Ethicon, Norderstedt, Germany). Lastly, skin closure was performed with a continuous intracutaneous suture (Monocryl 3.0, Ethicon, Norderstedt, Germany). Two suction drains (14 charriere) were inserted in each donor site. In the control group, patients received conventional wound management, which consisted of sterile adhesive strips (Leukosan Strip, BSN Medical, Hamburg, Germany) combined with a plaster (Leukomed, BSN Medical, Hamburg, Germany). In 2018, we changed our in-house wound management to CINPT due to promising reports in the literature. Since then, CINPT was placed on the suture line using a single layer of gauze, polyurethane foam, semi-occlusive transparent adhesive films, and a soft port (RENASYS-F, Smith & Nephews GmbH, Hamburg, Germany) (CINPT group), as shown in Figure 1. Continuous negative pressure was applied at 120 mmHg for 5 days (RENASYS TOUCH, Smith & Nephews GmbH, Hamburg, Germany). Then, patients from both groups received sterile adhesive strips (Leukosan Strip, BSN Medical, Hamburg, Germany). Absorbent cotton wool and elastic bandages were applied in both groups. Before discharge, all patients were provided with customized compression garments, which were worn until the third post-operative month. Mobilization was started on the first post-operative day. Drains were removed when the maximal fluid rate was below 30 mL per 24 h.

### 2.2. Statistics

The patient’s characteristics and outcomes were compared between the groups using the Fisher’s exact test and chi-square test for categorical variables, the two-sided unpaired *t*-test for normally distributed continuous variables, and Mann–Whitney U test for not normally distributed variables. Data are presented as frequencies for categorical variables, means and standard deviations for normally distributed variables, and medians and ranges for not normally distributed variables. All calculations were performed with the Prism 9.1.1 software (GraphPad Software, San Diego, CA, USA) and significance was set at *p* < 0.05.

## 3. Results

A total of 56 patients received 83 TMG flaps for breast reconstruction. A total of 35 patients with 53 TMG flaps were included in the control group and 21 patients with 30 TMG flaps were included in the CINPT group. Patient characteristics including medial preconditions and previous breast cancer therapy were comparable. The BMI was normal (<25 kg/m^2^) on average in both groups (control group 21.9 kg/m^2^ vs. CINPT group 23.9 kg/m^2^, *p* = 0.399). The prevalence of diabetes mellitus was <5% in the control group and 0% in the CINPT group. Bilateral TMG flap breast reconstructions were performed in >50% of the patients in both groups (control group 51.4% vs. CINPT group 52.4%, *p* = 0.944), with a high prevalence of genetic breast cancer predisposition seen in both groups. Comparative analysis of intraoperative details showed that the TMG flap width was significantly larger in the CINPT group (control group 7.0 cm vs. CINPT group 8.0 cm, *p* = 0.013). The operation time was similar in both groups (control group 260 min vs. CINPT group 302 min, *p* = 0.124). The total flap success rate was 79 out of 83 (95.2%), with no statistical difference between the groups. The follow-up time was significantly shorter in the CINPT group (*p* = 0.001). Patient characteristics and intraoperative characteristics are listed in Table 1 and Table 2.

The incidence of total surgical site complications on the medial thigh donor site was reduced in the CINPT group compared to the control, without statistical difference (control group 50.9% vs. CINPT group 30.0%, *p* = 0.064). The incidence of non-operative surgical site complications (control group 24.5% vs. CINPT group 10.0%, *p* = 0.107) and operative surgical site complications (control group 26.4% vs. CINPT group 20.0%, *p* = 0.511) was not statistically different between both groups. There was no difference in fluid drainage and time to drain removal. The in-hospital stay was significantly reduced in the CINPT group (control group 13 days vs. CINPT group 10 days, *p* = 0.030). Postoperative outcomes are summarized in Table 3.

**Figure 2:**
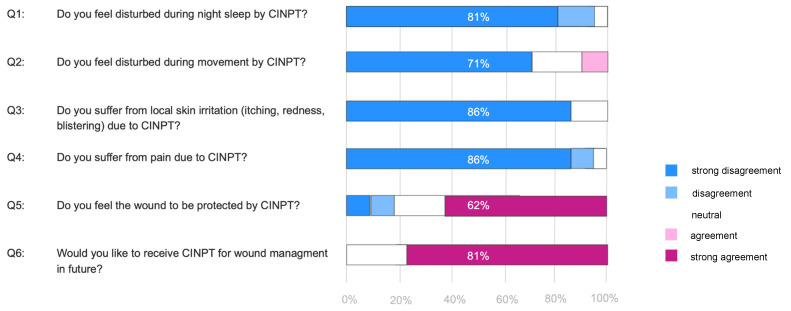
Patient survey. Responses to non-validated 5-point Likert scale questions (Q1–Q6) presented in color code. Color code on the right.

The survey disclosed positive patient perception applying CINPT for incision management on the medial thigh donor site. Limitations due to discomfort or noises during night sleep were negated in the CINPT group. Similarly, no skin irritations or local pain appeared in the CINPT group. Diminishment in movement was a minor complaint. The majority of patients stated they felt protected by CINPT application and would choose CINPT for incision management in the future. Patient perceptions are shown in Figure 2.

## 4. Discussion

The present study fails to provide significant evidence for the ability of CINPT to enhance incision healing on the donor site in TMG flap breast reconstruction. However, there was a trend toward lower donor site complications even though TMG flaps of larger sizes were harvested from the medial thigh. The in-hospital stay was significantly shortened. The patient survey disclosed that patients felt protected by prophylactic CINPT application and patient discomfort was excluded.

CINPT was designed for the management of closed incisions with reduced healing capacities. In an international multidisciplinary consensus paper, Willey et al. recommended the use of CINPT in patients with an individual high-risk profile (active smoking status, diabetes mellitus, BMI > 30 kg/m^2^, advanced age) or surgery-related high-risk profile (reoperation, prolonged operation time, incision tension forces) for surgical site complications, such as free flap breast reconstruction [21]. In 2018, Muller-Sloof et al. reported the ability of CINPT to lower the incidence of wound breakdown on the lower abdomen and posterior thigh donor sites in deep inferior epigastric perforator (DIEP) flap or profundal artery perforator (PAP) flap breast reconstruction compared to control in a small pilot trial (8% vs. 33%; *p* = 0.038) [17]. In 2020, our working group confirmed those preliminary findings and showed CINPT to significantly reduce surgical site complications (50.4% vs. 28.6%, *p* = 0.001) on the lower abdomen donor site in 300 DIEP and MS2-TRAM flap breast reconstructions [18]. In contrast, CINPT failed to show a statistically significant reduction in surgical site complications compared to conventional wound management in this study. The authors assume that this might be explained by the advantageous patient-related risk profile for surgical site complications in the patient collective qualifying for TMG flap for breast reconstruction. The patient characteristics in our study show that patients were young, healthy, and had a normal BMI on average. This parallels the results of a recent systematic review and meta-analysis including 843 TMG flap breast reconstructions outlining a young and normal weight patient profile. Surprisingly, the donor site complications on the medial thigh were shown to be low, in particular when compared to those in the patients with free flap breast reconstruction from the lower abdomen donor site [2,22]. It is well known that patient age correlates with wound healing and that advanced patient age represents an evident risk factor for surgical site complications due to relevant physiological changes such as loss of nutrition resources, reduction in stem cells and therefore reduced ability for regeneration. Obesity (BMI ≥ 30 kg/m^2^) is another serious key risk factor with multiple adverse health impacts [23]. In this context, Weitgasser et al. highlighted the average BMI value to be significantly lower in patients receiving double TMG flaps compared to double DIEP flaps for bilateral simultaneous breast reconstruction (TMG group 22.2 kg/m^2^ vs. DIEP group 28.8 kg/m^2^) with no significant relation to any other comorbidity or operation time [24]. Donor site complications were shown to be reduced and minor in the TMG flap patient collective (double DIEP patients 23.7% vs. double TMG patients 16.3%). This study discloses a trend toward reduced surgical site complications on the medial thigh donor site applying CINPT, even though TMG flaps of significantly larger size were utilized for free flap breast reconstruction. This is elemental, since one of the characteristic drawbacks of the TMG flap is its small size, which frequently necessitates lipo-fillings for volume augmentation [8]. Our preliminary results therefore cautiously support the application of CINPT to mitigate surgical site complications on the donor site in TMG flap breast reconstruction.

The harvest of the TMG flap creates an extensive dead space under the closed incision line, close to the main lymphatic collectors of the thigh, which may be prone to seroma formation. Previous studies have provided evidence that CINPT might reduce dead space and associated fluid collection at the donor sites in free flap microsurgery for other purposes [25,26]. In an animal model, Suh et al. showed the ability of CINPT to significantly reduce wound drainage following dead space closure by primary intention compared to a control [12]. This was explained by enhanced tissue perfusion and lymphatic clearance with reduction in edema and fluid collection. By contrast, in this study, daily fluid drainage and total fluid volume were slightly higher in the CINPT group, which might be due to the harvest of TMG flaps with significant larger size on average in this group. Additionally, CINPT had no effect on time to drain removal at the TMG donor site. Similarly CINPT had no effect on fluid drainage at the lower abdomen donor site in DIEP and MS2-TRAM breast reconstruction and ALT donor site compared to the control [16,18]. The authors assume that the evident effects of CINPT on the wound surface diminish in the deep soft tissue, which might explain the insufficient clinical effect in complex multi-layer wounds with strong fluid drainage in the deep soft tissue.

Surgical site complications regularly require medical or surgical intervention, extend the in-hospital stay, delay the recovery process, and raise healthcare costs substantially. In addition to this, surgical site complications reduce patients’ health-related quality of life and some studies even suggest associations with adverse cancer outcomes [27,28,29]. Therefore, uneventful and quick wound healing is of utmost importance for women undergoing breast reconstruction who survived the physical and emotional burden associated with mastectomy and adjuvant breast cancer therapy. In recent years, enhanced recovery pathways have been introduced in reconstructive breast surgery [30,31]. The concept is based on the minimization of pre- and postoperative surgical stress and early recovery aiming to optimize health outcomes. In this context patient perception, morbidity and length of in-hospital stay have become quality measures for best medical care. Our results indicate a significant reduction in in-hospital stay applying CINPT at the donor site in TMG flap breast reconstruction. The authors assume that in addition to our increasing experience with the TMG flap, prophylactic CINPT application contributed to the shortening of in-hospital stay due to enhanced wound healing and lower surgical site complications.

Innovations such as CINPT have been adopted to optimize outcomes in free flap breast reconstruction. However, it is unclear whether or not those innovations, which might be associated with additional health risk or substantial cost, are superior from a patient perspective. Even though CINPT is implemented as protective measure for wound healing in complex surgical procedures in various surgical disciplines, no validated assessment tool exists to survey patient-reported outcomes, and no study has investigated associated patient perceptions. This is the first study to exclude suggested adverse events of CINPT, including pain and skin irritations, which might appear due to direct contact with polyurethane foam, discomfort during night sleep, and limitations in mobility. In contrast, our patient survey highlighted positive patient perceptions and disclosed that patients felt protected by prophylactic CINPT application.

Surgical site complications are associated with substantial costs for the health care system. Gabriel et al. reported on potential cost savings by applying CINPT in immediate implant-based breast reconstruction [13]. However, commercial CINPT products are expensive and regularly do not fit on complex surgical sites with non-linear incision lines or limited surrounding tissue. To ensure proper CINPT function and patient comfort, we use a custom-made in-house standardized CINPT which prevents adverse effects to the skin, loss of CINPT adherence, and loss of airtight seal. In the unlikely event of minor air leakage or the weakening of CINPT adherence, semi-occlusive transparent adhesive films is applied to enhance the system. Our CINPT is an off-label use of the material and portable device for negative pressure therapy for open wounds. Previous studies have confirmed its successful use on other donor sites in free flap microsurgery [16,18]. Additionally, new designs of negative pressure therapy, such as closed incision negative pressure therapy for incisions and surrounding soft tissue, rely on the safe application and off-label use of conventional products for negative pressure therapy for open wounds [32].

Based on the positive results of this study, CINPT application will be used for wound management on the donor site in TMG flap breast reconstruction in future.

The present study has some limitations. The number of patients was small, though still comparable to other similar studies in the literature [17,20]. This might lead to a type 2 statistical error, explaining the lack of statistical evidence for the effectiveness of CINPT in TMG flap breast reconstruction despite the observed differences between both study groups. In addition, an economic analysis on the use of CINPT is lacking. There is need for prospective randomized controlled trials to validate our preliminary results and to establish best practice guidelines.

## 5. Conclusions

Evidence exists for the superiority of CINPT compared to conventional wound management on the donor site in patients undergoing free flap breast reconstruction from the lower abdomen. However, the present study fails to provide compelling evidence for CINPT to enhance incision healing on the donor site in the patients undergoing TMG flap breast reconstruction. Our preliminary data still show a trend towards lower surgical site complications on the medial thigh donor site and a shortened in-hospital stay. This is the first study to disclose that patients feel protected by prophylactic CINPT application and patient discomfort could be excluded.

## Figures and Tables

**Table 1:** Patient characteristics.

	Control Group	CINPT Group	*p*-Value
**Patients**, N	35	21	NA
**TMG flaps**, N	53	30	NA
**Age** [years], median (R)	45 (24–66)	42 (30–77)	0.334
**BMI** [kg/m^2^], median (R)	21.9 (15.6–32.5)	23.9 (18.2–33.0)	0.399
**Breast cancer**, N	24 (68.5%)	18 (85.7%)	0.151
**Genetic breast cancer predisposition**, N	12 (34.3%)	10 (47.6%)	0.322
**Risk Factors**, N			
Diabetes mellitus	1 (2.8%)	0 (0%)	0.999
Obesity (BMI ≥ 30kg/m^2^)	4 (11.4%)	1 (4.8%)	0.397
Active smoker	7 (19.0%)	4 (20.0%)	0.999
Preoperative chemotherapy	13 (37.1%)	8 (38.1%)	0.943
Preoperative radiotherapy	13 (37.1%)	13 (61.9%)	0.072
**Breast reconstruction laterality**			
Unilateral	17 (48.6%)	10 (47.6%)	0.944
Bilateral	18 (51.4%)	11 (52.4%)	0.944
**Follow-up** (months), median (R)	70 (40–144)	17.5 (4–47)	**0.001** *

CINPT, closed incision negative pressure therapy; N, number; NA, not applicable; BMI, body mass index; R, range; * significant difference (*p* < 0.05).

**Table 2:** Intraoperative characteristics.

	Control Group	CINPT Group	*p*-Value
**Donor sites**, N	53	30	NA
**Flap width** [cm], median (R)	7.0 (5–8)	8.0 (6–10)	**0.013** *
**Operation time** [minutes], median (R)	260 (160–832)	302 (194–651)	0.124

CINPT, closed incision negative pressure therapy; N, number; NA, not applicable; R, range; * significant difference (*p* < 0.05).

**Table 3:** Postoperative outcomes.

	Control Group	CINPT Group	*p*-Value
**Donor sites**, N	53	30	NA
**SSC**, **total**, N (%)	27 (50.9%)	9 (30.0%)	0.064
**Non-operative SSC**, N (%)	13 (24.5%)	3 (10.0%)	0.107
Delayed wound healing	5 (9.4%)	2 (6.7%)	>0.999
Seroma	5 (9.4%)	1 (3.3%)	0.410
Hematoma	1 (1.9%)	0	>0.999
Wound infection	1 (1.9%)	0	>0.999
**Operative SSC**, N (%)	14 (26.4%)	6 (20.0%)	0.511
Seroma	2 (3.8%)	1 (3.3%)	>0.999
Skin necrosis	3 (5.7%)	3 (10.0%)	0.662
Hematoma	2 (3.8%)	1 (3.3%)	>0.999
Wound infection	2 (3.8%)	1 (3.3%)	>0.999
Wound dehiscence	5 (9.4%)	0 (0.0%)	0.313
**Drain removal** [postoperative day], median (R)	5 (3–13)	6 (4–13)	0.105
**Drain delivery rate** [mL], median (R)			
Total	295 (50–1875)	430 (20–1390)	0.125
Day 1	100 (0–470)	100 (0–310)	0.864
Day 2	70 (0–350)	78 (0–290)	0.586
Day 3	50 (0–280)	70 (0–340)	0.274
Day 4	28 (0–370)	50 (0–150)	0.063
Day 5	0 (0–220)	45 (0–150)	0.077
**In-hospital stay** [days], median (R)	13 (8–29)	10 (7–21)	**0.030** *

CINPT, closed incision negative pressure therapy; SSC, surgical site complications; N, number; NA, not applicable; SD, standard deviation; R, range; * significant difference (*p* < 0.05).

## Data Availability

The data presented in this study are available on request from the corresponding author. The data are not publicly available due to ethical, legal, and privacy issues.
